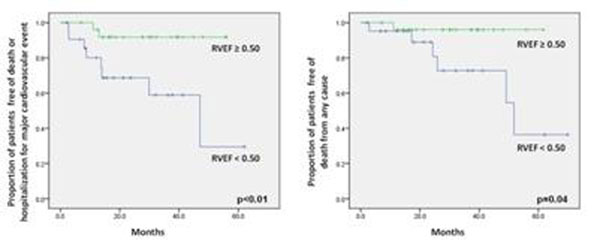# Right ventricular dysfunction predicts clinical outcomes following cardiac resynchronization

**DOI:** 10.1186/1532-429X-13-S1-O104

**Published:** 2011-02-02

**Authors:** Francisco Alpendurada, Kaushik Guha, Rakesh Sharma, Sanjay Prasad

**Affiliations:** 1 Royal Brompton Hospital, London, UK

## Introduction

Cardiac resynchronization therapy (CRT) is an established treatment for patients with advanced heart failure (HF). However, a significant proportion of patients do not gain benefit from CRT, the reasons for which are unclear. Despite an established predictive role in HF, the significance of right ventricular (RV) dysfunction in gauging clinical benefit from CRT has not been elucidated. Cardiovascular magnetic resonance (CMR) is an important tool in the assessment of HF and is considered the gold-standard in estimating RV function. We used this technique to assess the impact of RV dysfunction on clinical outcomes in HF patients receiving CRT.

## Methods

We evaluated 48 consecutive patients attending a heart failure pacing clinic who had a CMR study within 6 months of CRT implantation. Clinical, biochemical, ECG and imaging data were collected. CMR parameters included biventricular function and myocardial scar assessed by gadolinium enhancement. The primary end-point was a composite of death from any case or unplanned hospitalization for a major cardiovascular event. Patients were also evaluated for response to CRT. This was defined as an improvement of left ventricular ejection fraction (LVEF) >5% at 12 months after device implantation.

## Results

The mean age was 64.5±12.7 years. HF was ischemic in 42% of patients, and 85% were in NYHA class III/IV at the time of implantation. Atrial fibrillation/flutter was found in 27% of patients. The mean LVEF estimated by CMR was 27 ± 8%, while the median RVEF was 52% (interquartile range 35-63%). The mean tricuspid annular plane systolic excursion (TAPSE) was 14.0 ±6.0 mm, and the mean pulmonary artery pressure (determined by echocardiography) was 36.9 ±10.4 mmHg. Ten patients (21%) met the primary end-point over a mean follow-up period of 28.6 months, and 21 out of 44 patients (48%) were considered responders to CRT. On time-to-event analysis, only atrial fibrillation (HR 4.8, p=0.02) and RV dysfunction, either by reduced RVEF (HR 0.96, p=0.01) or TAPSE (HR 0.80, p<0.01) were independent predictors of the primary end-point. Atrial fibrillation and low RVEF were the only independent predictors of all-cause mortality (log-rank p=0.03 and 0.04, respectively). Coronary artery disease, as well as impaired RV function, emerged as independent predictors of non-response to CRT.

## Conclusions

Right ventricular dysfunction is an independent predictor of adverse clinical outcome as well as response to CRT. Routine assessment of the right ventricle may be of benefit for selecting patients for CRT implantation.

**Figure 1:**